# Clinical History and Colliquative Myocytolysis Are Keys to the Diagnosis of Shoshin Beriberi

**DOI:** 10.1155/2014/506072

**Published:** 2014-05-07

**Authors:** Toshiki Kuno, Hiroshi Nakamura, Yutaka Endo, Kohei Saito, Hiroyuki Yamazaki, Hiroyuki Motoda, Yohei Numasawa, Kazuhiko Shimizu, Toshiyuki Takahashi

**Affiliations:** ^1^Department of Cardiology, Ashikaga Red Cross Hospital, 284-1 Yobe-cho, Ashikaga City, Tochigi 326-0843, Japan; ^2^Department of Community Health and Medicine, School of Medicine, Yamaguchi University, Yamaguchi, Japan; ^3^Department of Clinical Laboratory and Pathology, Ashikaga Red Cross Hospital, Tochigi, Japan

## Abstract

Cardiovascular beriberi presents as either the fulminant (Shoshin beriberi) or chronic form. Shoshin beriberi is a rare disease that may lead to a fatal outcome if the patient does not receive appropriate treatment. In the present report, we describe the case of a 66-year-old man presenting with leg edema and dyspnea at rest. Clinical presentations were nonalcoholic Shoshin beriberi and lactate accumulation; however, clinical improvement was observed after the administration of thiamine. His pretherapy thiamine level (2.1 **μ**g/dL) was consistent with a diagnosis of beriberi. Based on the findings of the present case, we believe that a diagnosis can be made in patients with a clinical history that is consistent with that of Shoshin beriberi, combined with low thiamine levels, lactate accumulation, and colliquative myocytolysis. *Learning Objective.* Shoshin beriberi is often misdiagnosed because of its rarity; a detailed clinical history and characteristic myocardial histopathology changes may be useful for making a definite diagnosis.

## 1. Introduction

Thiamine (vitamin B1) is a key cofactor of important metabolic enzymes, and, therefore, thiamine deficiency (TD) can cause alterations in heart muscle metabolism. Typical wet beriberi (cardiac beriberi) cases show severe metabolic acidosis, high output biventricular failure, and markedly low systemic vascular resistance [1]. TD can also cause three other clinical syndromes: dry beriberi (peripheral neuropathy), Wernicke-Korsakoff encephalopathy, and Leigh's syndrome (subacute necrotizing encephalopathy) [2, 3]. TD neuropathy is distinct from alcoholic neuropathy [4], and wet beriberi presents with varying degrees of cardiovascular involvement. In general, dietary thiamine can be obtained through the consumption of pork, soybeans, unpolished rice, and eel. According to previous Japanese studies, dietary imbalances, chronic alcoholism, and a history of gastrectomy are the three major causes of TD [2–4].

Cases of acute, or fulminant, wet beriberi are occasionally called “Shoshin beriberi”; in Japanese, “Sho” indicates acute damage, and “shin” indicates the heart [5]. Shoshin beriberi is characterized by hypotension, tachycardia, and lactic acidosis. If the patient does not receive appropriate treatment because of misdiagnosis, the patient may die within hours due to circulatory collapse and pulmonary edema. Morphologically, myocardial necrosis and colliquative myocytolysis are the histologic hallmarks of this rare, acute clinical entity [6]. This condition often remains undiagnosed. Therefore, in the present report, we describe a case of nonalcoholic Shoshin beriberi and demonstrate that the clinical history and pathognomonic and histopathological changes in the myocardium are keys to its diagnosis.

## 2. Case Presentation 

A 66-year-old man was admitted to our hospital in April 2013 with a 1-week history of leg edema and dyspnea at rest. He did not have a medical history or a history of alcoholism. Because he was unable to walk due to lumbosacral strain, he had been consuming only convenience foods for the previous 2 months. Upon admission, a physical examination revealed irregular tachycardia, a blood pressure of 96/78 mmHg, high jugular venous pressure, and coarse crackles in both lungs. His skin was cold and his legs were swollen, suggestive of heart failure; he had palpable pedal pulses. An abdominal examination showed a distended abdomen, suggestive of ascites, and his bowel sounds were reduced. His orientation in terms of the time and place was normal, and he did not report any numbness in his lower limbs. In addition, he did not report any weakness in his arms or legs but experienced a feeling of tenderness on his back. He had reduced patellar reflexes and his Achilles tendon reflexes were absent. An electrocardiogram showed atrial fibrillation with a rapid ventricular response (heart rate, 140 beats/min) without ST-T changes, right axis deviation, or low voltage. A chest radiograph showed cardiomegaly, pleural effusion in half of each lung, and lung congestion; an echocardiogram revealed a left ventricular ejection fraction of 23% (Modified Simpson's method; normal range: >50%), with diffuse hypokinesis of the left ventricle. The left ventricle end diastolic and end systolic diameters were 50 and 45 mm (normal ranges: 48 ± 4 mm and 34 ± 4 mm), respectively.

The patient's arterial blood gases demonstrated a pH of 7.30, oxygen partial pressure of 61.1 mmHg, carbon dioxide partial pressure of 31.6 mmHg, bicarbonate level of 13.0 mmol/L, and lactate level of 4.2 mmol/L (normal range: 0.5–1.6 mmol/L). Further laboratory investigation showed a creatine phosphokinase level of 1035 U/L, with a muscle brain fraction of 56 U/L, troponin-T level of 0.055 ng/mL, C-reactive protein level of 1.81 mg/dL, and B-type natriuretic peptide level of 1566 pg/mL. Thyroid function was normal.

Cardiac beriberi was suspected based on the patient's clinical history and laboratory findings; therefore, we immediately administered 50 mg of thiamine and started continuous infusions of furosemide (40 mg/day), carperitide (0.025 *μ*g/kg/min), and thiamine (100 mg/day). On the second day, the patient's bicarbonate level had improved to 21.6 mmol/L, and his lactate level had decreased to 1.6 mmol/L; his clinical condition improved, thereafter. The thiamine dosing resulted in a thiamine level of 2.1 *μ*g/dL (normal range: 2.6–5.8 *μ*g/dL). On day 11, brain magnetic resonance imaging was performed, which did not show any evidence of Wernicke encephalopathy.

**Figure 1:**
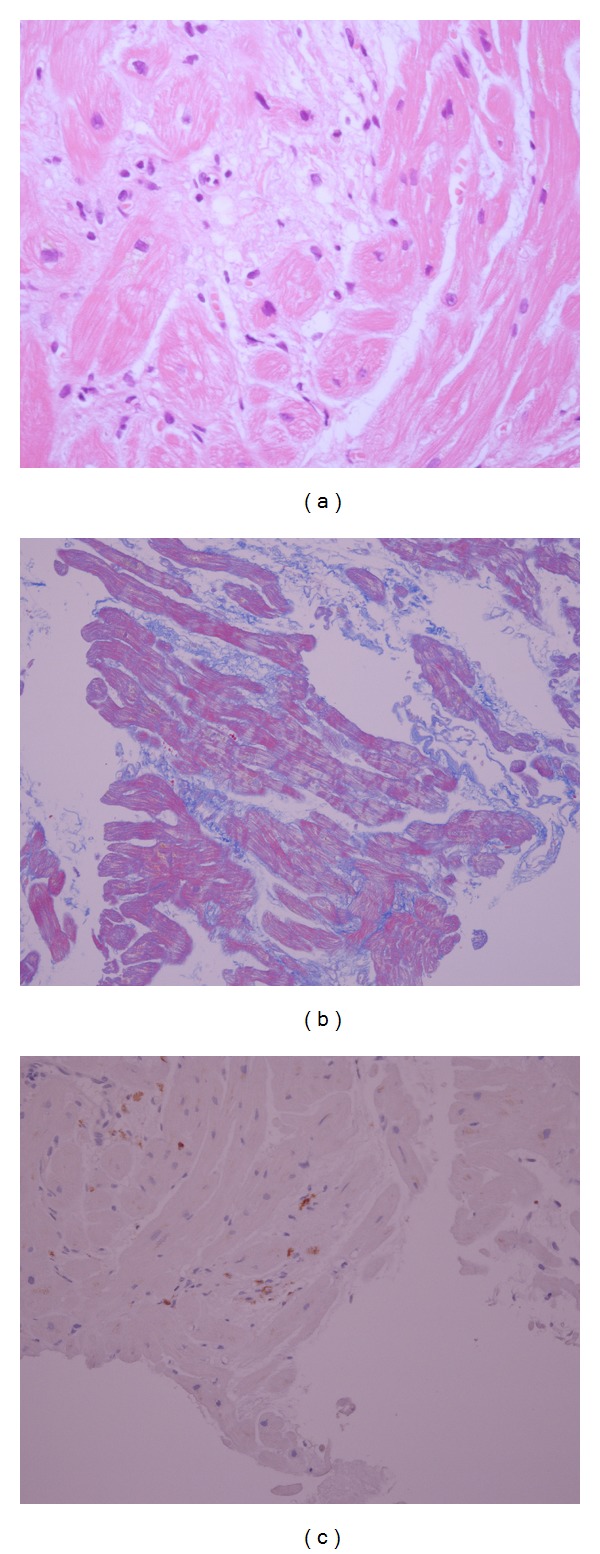
Myocardial biopsy histopathology. (a) Grade 2 colliquative myocytolysis and perinuclear disappearance of myofibrils with intramyocardial edema appearing as an empty sarcolemmal tube. Hematoxylin-eosin stain, ×400. (b) Interstitial fibrosis is evident. Azan staining, ×200. (c) Mild cell infiltration in the myocardium. Immunostaining for CD 45 antigens; hematoxylin-eosin stain, ×200.

On hospital day 23, a coronary angiogram revealed normal coronary arteries, and right heart catheterization demonstrated a pulmonary artery pressure of 38/18 mmHg, mean pulmonary wedge pressure of 21 mmHg (normal range: <12 mmHg), and cardiac output of 2.7 L/min, with a cardiac index of 1.9 L/min/m^2^ (normal range: >2.2 L/min/m^2^) (thermodilution method). A right ventricular endomyocardial biopsy was performed, which indicated interstitial fibrosis, mild myocyte hypertrophy, and mild cell infiltration (Figure 1).  Figure 1(a) shows grade 2 colliquative myocytolysis and the perinuclear disappearance of myofibrils, with intramyocardial edema presenting as an empty sarcolemmal tube.  Figure 1(b) shows interstitial fibrosis in the myocardium with Azan staining. Mild cell infiltration was seen with CD 45 staining (Figure 1(c)).

We diagnosed the patient with Shoshin beriberi based on the combination of the clinical history, low thiamine level, lactate accumulation, and colliquative myocytolysis of the myocardium. On hospital day 38, he was discharged without any signs of heart failure. After 24 days, a repeat echocardiogram showed a left ventricle ejection fraction of 39% with diffuse hypokinesis of the left ventricle. He was followed for 9 months without episodes of heart failure.

## 3. Discussion

The major causes of TD are dietary imbalance, chronic alcoholism, and a history of gastrectomy [2, 3]. Other causes include poor oral intake; inadequate provision of thiamine in enteral or parenteral nutrition therapy; malignancies; AIDS; pregnancy and lactation; hyperthyroidism; renal failure, particularly in those receiving hemodialysis; loop diuretic use; systemic infections; advanced age; diabetes mellitus; and bariatric surgery [7].

TD causes a severe reduction in pyruvate dehydrogenase activity, which prevents the conversion of pyruvate into acetyl-coenzyme A. This decrease in acetyl-coenzyme A results in a deficiency of reduced nicotinamide adenine dinucleotide, which eventually results in decreased cellular adenosine triphosphate levels [8]. The resultant impaired myocardial energy production might reasonably alter ventricular performance. In addition, pyruvate and lactate cause intense vasodilatation due to the presence of peripheral arteriovenous shunts in the skeletal muscles. In the classic form, these pathophysiological mechanisms cause high output heart failure and marked edema. However, in some cases, fulminating low-output heart failure has been noted [5].

The diagnosis of Shoshin beriberi is always difficult owing to the lack of laboratory facilities for detection and the lack of the requisite tests in emergency settings and because TD is not usually considered in patients with acute heart failure. Shoshin beriberi, if not rapidly diagnosed and promptly treated, may result in rapid hemodynamic collapse and death [5]. In some autopsy cases, myocardial examination has shown extended colliquative myocytolysis [6]. Colliquative myocytolysis is used to define the progressive loss of myofibrils in conjunction with intramyocellular edema [9]. Colliquative myocytolysis is the histological hallmark of congestive heart failure, including acute myocardial infarction, and this pathology is indicative of a secondary, nonischemic complication involving the subendocardial myocardium that causes infarct necrosis. Myocardial necrosis and colliquative myocytolysis are histologic characteristics of acute Shoshin beriberi [6].

In the differential diagnosis, the possibility of myocarditis should also be considered in cases of infiltration of inflammatory cells; however, we could differentiate this case from myocarditis based on the following points. First, the invasion of inflammatory cells was mild. Second, these cells were mainly lymphocytes, rather than macrophages. Third, the location of inflammation was nonspecific. In the case of infection, the inflammation would primarily involve the myocardium and vessels. Fourth, the interstitial fibrotic change was diffuse and independent of the inflammatory cells. Finally, this case did not demonstrate evidence of ST-T changes in the electrocardiogram or the elevation of the creatine phosphokinase muscle brain fraction and troponin T levels. Therefore, a myocardium biopsy would provide an auxiliary diagnosis, which would enable the differentiation of this condition from other disorders.

In conclusion, we report a case of nonalcoholic Shoshin beriberi. Although this condition is often misdiagnosed because of its rarity, a detailed clinical history evaluation and an assessment of the characteristic myocardial histopathology changes may be useful in making a definite diagnosis.
